# Good Food and Food Poisoning

**Published:** 1917-04

**Authors:** S. R. Klein

**Affiliations:** Director Norwich Research Laboratory, Formerly Professor Fordham University Medical School, Norwich, Conn.


### Journal-Record of Medicine

Successor to Atlanta Medical and Surgical Journal, Established 1855
and Southern Medical Record, Established 1870

**OWNED BY THE ATLANTA MEDICAL JOURNAL COMPANY**

PUBLISHED MONTHLY

*Official Organ Fulton County Medical Society, State Examing Board, Atlanta, Birmingham and Atlantic Railroad Surgeon's Association, Chattahoochee Valley Medical and Surgical Association, Etc.*

A. W. STIRLING, M. D., C. M., D. P. H.; J. S. HURT, B. Ph., M.D
GEO. M. NILES, M. D., W. J. LOVE, M. D., (Ala.); Associate Editors

E. W. ALLEN, Business Manager

**COLLABORATORS**

W. F. WESTMORELAND, M. D., General Surgery
F. W. McRAE, M. D., Abdominal Surgery
ALLEN H. BUNCE, Medical Societies
E. B. BLOCK, M. D., Diseases of the Nervous System
MICHAEL HOKE, M. D., Orthopedic Surgery
CYRUS W. STRICKLER, M. D., Legal Medicine and Medical Legislation
E. C. DAVIS, A. B., M. D., Obstetrics
E. G. JONES, A. B., M. D., Gynecology
ALLEN H. BUNCE, M. D., Pathology and Bacteriology
R. T. DORSEY, JR., B. S., M. D., Medicine
L. M. GAINES, A. B., M. D., Internal Medicine
GEO. C. MIZELL, M. D., Diseases of the Stomach and Intestines
L. B. CLARKE, M. D., Pediatrics
EDGAR PAULLIN, M. D., Opsonic Medicine
THEODORE TOEPEL, M. D., Mechano Therapy
E. G. BALLENGER, M. D., Diseases of the Genito-Urinary Organs

VOL. LXIV. ATLANTA, GA., APRIL, 1917. NO. 1

### GOOD FOOD AND FOOD POISONING.

By S. R. Klein, M. D., Ph. D., M. A., Director Norwich Research Laboratory, Formerly Professor Fordham University Medical School, Norwich, Conn.

Food must be accustomed to the climate and habit of the people. Under warm climate animal food should be diminished and a more liberal allowance of vegetable food taken. The latter supplies the necessary constituents in a less stimulating form and one more suited to a climate in which congestion of

13**S

the animal viscera is especially apt to occur. Carbohydrates furnish energy with a moderate production of internal heat, hence they are very valuable in the form of maize, rice, lentils, as they contain less nitrogen. Sugar is excellent when energy is to be liberated rapidly with the least tax upon the digestive system. During the present European war cavalry horses recovered health and energy when their coarse grass or hay was sprinkled with molasses or sweetened water. In grain eating populations starchy constituents bulk too largely and require to be supplemented by a little animal food rich in oil, or seeds rich in albuminates or oil, such as ground nuts, or by adding other pulses less rich in oil and supplying the requisite amount of oil separately.

El Gofio is a food which has been praised for use in warm climates. It is manufactured from different flours, including those obtained from wheat, maize, barley, white lupine, rye, chick peas and beans. When prepared with milk and eggs it gives an excellent nourishment. Spirits and beer are positively detrimental to health, light wines, white or red, do least harm. Champagne taken after excessive fatigue, about sunset, is perhaps the safest form of alcoholic beverage. It should not be taken with meals, but only on reaching home after a fatiguing march, or long exposure to wet.

Tea—as a stomachic tonic and as a safe way of introducing fluid to the system, would seem beneficient and hygienic. It was introduced by the Chinese, owing to the calamities arising from drinking unboiled water. The Chinese do not drink tea during their meal, but after the meal is finished. The pernicious system of drinking tea during a meal is one peculiar to British people and the habit is fraught with many dyspectic troubles. The best China tea, prepared by pouring boiling water over the leaves and immediately pouring the water off the leaves, is a wholesome fluid, calculated to aid digestion, especially when taken after the meal is finished. Tea taken with animal food, as eggs, fish, or flesh, is a certain means of producing dyspepsia, for when the tea is ''drawn'' for a long time, and when the tea used is of inferior quality, the tannic acid of the decoction, uniting with the albumen of the animal tissues, produces a leathery compound which no gastric juice can penetrate and digest· Coffee—2 or 3 mouthfuls of after a meal, is an aid to digestion; taken in quantity—breakfast cupfuls—it is an impediment to digestion, and diluted with half milk and taken with a meal of eggs, fish or meat is still more so. Tobacco smoked in moderation soon after meal the deleterious effects of tobacco are infinitesimal. When indulged in to excess, six to eight cigars or fifteen—twenty cigarettes, or one ounce of pipe tobacco, it is an injurious cardiac depressant.

The most dangerous tinned foods are those containing much moisture, i. e·, milk, salmon, lobster and mixtures of meat and vegetables. The more acid foods such fruit, jams, and vegetables, are more liable to take up metals from the tins. The simpler the preparation the better it stands the effects of climate and heat. Apparent bulging may be due to the tins being dented. A good tin of meat has usually slightly concave ends owing to a partial vacuum forming during the process of sterilization. Resoldering should be looked for. As a rule, two holes are made in one end of the tin to permit steam to escape. Resoldering, or the presence of a third or more soldered holes points to puncture to allow gas to escape. Dented tins, if otherwise fit, should be issued early, as they are apt to rust and perforate on keeping. On opening certain tins, i. e., of marmalade, rhubarb, tomato soup, etc., a blackened appearance may be noticed. This is due to the action of the vegetable acids on the tin plating and if slight, and there is no evidence of fermentation as evidenced by minute gas bubbles, may be neglected. Decomposition may result from incomplete sterilization, or incomplete sealing of the tin. Bulged tins may be tested by puncturing them under water to test for the escape of gas· In some cases a little gas will escape from tins containing perfectly sound meat, owing to incomplete exhaustation during the process of sterilization, but which, being sterile, is of no real consequence and amounts to, as a rule, only about 1 c. c. or so. Professor Eber's test for the decomposition of meat consists of one part sulphuric ether, one part pure H C L and 3 parts ethylic alcohol is placed in a test tube or other suitable vessel. The material to be examined is smeared on the end of a glass rod, which is dipped below the surface of the reagent but is not allowed to touch the side. If ammonia be present, a cloudiness appears or fumes may be given off.

S. R. KLEIN, M. D.

